# Development and usability of educational material about workplace particulate matter exposure

**DOI:** 10.1186/s12889-021-10197-x

**Published:** 2021-01-22

**Authors:** T. A. M. Stege, J. F. B. Bolte, L. Claassen, D. R. M. Timmermans

**Affiliations:** 1 National Institute for Public Health and the Environment (RIVM), PO Box 1, 3720 BA Bilthoven, The Netherlands; 2 Smart Sensor Systems group, Faculty of Technology, Innovation, and Society, The Hague University of Applied Sciences, Rotterdamseweg 137, 2628 AL Delft, The Netherlands; 3 Department of Public and Occupational Health, Amsterdam Public Health research institute, Amsterdam University Medical Center, PO Box 7057, 1007 MB Amsterdam, The Netherlands

**Keywords:** Particulate matter, Risk communication, Occupational exposure, Educational folder

## Abstract

**Background:**

Particulate matter (PM) exposure is an important health risk, both in daily life and in the workplace. It causes respiratory and cardiovascular diseases and results in 800,000 premature deaths per year worldwide. In earlier research, we assessed workers’ information needs regarding workplace PM exposure, the properties and effects of PM, and the rationale behind various means of protection. We also concluded that workers do not always receive appropriate risk communication tools with regards to PM, and that their PM knowledge appears to be fragmented and incomplete.

**Methods:**

We considered several concepts for use as an educational material based on evaluation criteria: ease of use, costs, appropriateness for target audiences and goals, interactivity, implementation issues, novelty, and speed. We decided to develop an educational folder, which can be used to inform employees about the properties, effects and prevention methods concerning PM. Furthermore, we decided on a test setup of a more interactive way of visualisation of exposure to PM by means of exposimeters. For the development of the folder, we based the information needs on our earlier mental models-based research. We adjusted the folder based on the results of ten semi-structured interviews evaluating its usability.

**Results:**

The semi-structured interviews yielded commentaries and suggestions for further improvement, which resulted in a number of alterations to the folder. However, in most cases the folder was deemed satisfactory.

**Conclusion:**

Based on this study, the folder we developed is suitable for a larger-scale experiment and a practical test. Further research is needed to investigate the efficacy of the folder and the application of the exposimeter in a PM risk communication system.

**Supplementary Information:**

The online version contains supplementary material available at 10.1186/s12889-021-10197-x.

## Background

Particulate matter (PM) is an important exposure risk in society [38], as well as in various workplaces, for example in roadwork companies [22, 36]. People in these companies regularly inhale the small particles of PM, especially ones with a diameter smaller than 2.5 μm (PM_2.5_), resulting in potential detrimental health effects [17, 31]. These effects may include cardiovascular and respiratory diseases, such as lung cancer and bronchitis [17]. The effects of PM exposure are estimated to cause around 800,000 annual deaths worldwide [2]. Protection against PM involves such measures as sprinkling water, respirators, filtering systems and ventilation [18, 35]. Research shows that personal protection against PM, mainly in the form of various types of respirators, has a profound effect on PM reduction; however, not all workers that are exposed to PM use them appropriately [21].

The protection motivation theory, or PMT [27], distinguishes two processes that influence the motivation to protect against risk. These processes are threat appraisal, which is the perceived expected risk subtracted by the benefits of risky behavior, and coping appraisal, which is the perceived efficacy of protective behavior subtracted by its cost. In general, a higher threat appraisal and a higher coping appraisal lead to a higher tendency to protect oneself against a certain risk. However, in some cases, higher threat appraisal might be counterproductive, and cause people to ignore the message [16, 28]. This may be explained by fear eliciting a maladaptive response as people avoid the risk communication message rather than the risk itself [27]. However, not all researchers agree that these counterproductive effects exist, and some say there is simply a cap on the benefits of threat appraisal [32]. Either way, these factors should be taken into account when designing a risk communication material.

In earlier research [30], we assessed specific information needs of employees in roadwork companies concerning PM exposure. We did this by means of a mental models approach. Mental models can be defined as *“personal, internal representations of external reality that people use to interact with the world around them”* [19]. In risk communication and risk perception research, the mental models approach is a systematic way to map these representations of a risk (that is about sources, properties, exposure, effects and mitigation options), and to contrast the representations of various groups of people, such as experts and non-experts [8, 23]. The concepts of threat appraisal and coping appraisal from the PMT model mentioned earlier resemble the various aspects of mental models of risk. That is, beliefs about sources, hazardous properties, exposure and effects of a certain risk are closely linked to threat appraisal, and beliefs about mitigation methods can be linked to coping appraisal.

After mapping the mental models of various groups, the differences between them are used to identify information needs in risk communication. This way, risk communication can alleviate common misconceptions and answer common questions about the subject matter [29]. The mental models approach has been used in a wide array of risk-related subjects resulting in suitable risk communication tools, ranging from flood prevention to cigarette smoking [26].

The mental models approach in our previous study [30] yielded a scientific and an employee mental model for particulate matter. The scientific mental model was extracted from literature on PM, and corroborated by interacting with experts in the field. The employee mental model was erected after conducting 22 semi-structured interviews with employees in the roadwork sector. An overview of the main differences between both of these mental models can be found in Table 1.

**Table 1:** Overview of discrepancies between scientific and employee mental models [30]

Subject	Scientific mental model	Employee mental model
Properties	PM is usually invisible	It is unclear whether PM is visible or not
	It is not possible to smell PM	It may be possible to smell PM
	Black carbon, metals, silicium and rubber are important constituents of PM	–
	Particle size is most often defined in terms of PM_10_, PM_2.5_ and PM_0.1_	–
	PM mostly consists of solid particles, but may also include liquid particles or semi-volatile compounds.	–
Sources	–	Sand and dirt roads cause PM
	There are natural sources of PM, such as sea salt, which don’t cause adverse health effects.	–
Effects	–	PM exposure may cause headaches and nausea
	PM exposure is associated with cardiovascular disease, even more so than with respiratory disease	(Almost) no mention of cardiovascular disease; only attention for respiratory diseases
	PM causes about 800,000 annual premature deaths worldwide.	–
	PM is also an environmental risk (for example due to acid rain or nutrient depletion).	–
Prevention	There is an occupational hygiene strategy that involves a four-level hierarchical model, which should be followed to reduce PM exposure.	There are a large number of prevention methods (sprinkling water, respirators, …) that could be used to reduce PM exposure.
Education and empowerment	A viable education system improves safety culture and willingness to protect against (exposure) risks.	The current education system could be improved; it is often too ritualistic and repetitive, and not everyone is involved with the process.

One question that comes to mind is how to convey quantitative risk information about health effects and exposure. Research recommends using a so-called ‘X in 100’ format to convey the potential health effects in a population [34, 37], as percentages alone may confuse the reader and lead to false interpretations. These ‘X in 100’ formats are generally preferred by respondents to similar formats such as ‘1 in X’ [37]. In general, visually enhancing risk information with graphs tends to be more effective than simply providing verbal or numeral information [15, 20]. Our own experience in an earlier study was that employees in the roadwork branch tend to find graphs about workplace exposure interesting and insightful [6].

Nevertheless, graphs can also be inadvertently misleading; an example of this involves participants judging a cardiovascular risk from a bar chart as relatively low compared to 100%, even though experts would say that the risk is quite high [10]. Therefore, it is imperative to choose an appropriate format. Specifically, when considering the number of individuals affected in a population, a ten-by-ten matrix of human icons may be used to convey a percentage [20]. Visualizations such as these help reduce several biases, including framing effects and denominator neglect [34], although their effectiveness is not explained by an improvement of exact knowledge about the risk; only ‘gist knowledge’ appears to be increased [14].

In our situation, we would like to give a rough but accurate estimate of the health risk of PM, in order to induce an accurate representation of the risk. Exact numbers for the amount of work-related deaths due to PM are unknown, as the earlier mentioned 800,000 deaths per year worldwide applies to all people in general, without any indication how many of these deaths are work-related [2]. There are studies that estimate the burden of disease for workplace exposure, although these studies generally only take forms of cancer into account, not other adverse health effects; one estimate states that 10% of all lung cancer in males and 5% in females can be attributed to work, which amounts to a worldwide DALY loss of 969.000 [13]. Another estimate can be generated from a factsheet about hazardous substances at work [3], which mentions that 1 million people in the Netherlands are exposed to one or more hazardous substances at work, and 3000 of those people die each year. Although these may be various types of substances and not just PM, the most important substances mentioned are all a form of PM, such as diesel emission [18] or quartz [36].

In our earlier research [30], we found that interventions in the workplace about exposure risks tend to focus on a specific substance. Work safety meetings about, for example, minuscule quartz or wood particles appear to be more commonplace than work safety meetings about PM in general. We decided to broaden the scope to PM in general for this study for several reasons. Many of these types of PM are caused by similar acts, such as sawing and drilling. Although the substances that are a part of PM differ in their toxicity, the effects on the human body are still explained for a significant part by their particle size as well, as the small particles enter into the lungs and blood stream [17]. For these reasons, we decided to develop an educational material with a focus on PM in general, mentioning various types and sources of PM in the material itself. It should be noted that, because of this decision, our educational material should be used with the goal of general health promotion in mind, in smaller-scale work safety meetings. For more in-depth education on a specific subject or a specific content of particulate matter, additional educational material on potential adverse health effects may be needed.

In this study, we considered the aforementioned recommendations about contents as a basis for developing and testing new educational material about workplace PM exposure. Next, we will present the method by which we developed this new educational material. Furthermore, we consulted experts on risk communication, particulate matter, or both, as well as workers that may be exposed to PM, in order to inquire about the usability of our educational material. The question we would like to answer is, ‘How do stakeholders perceive the usability of a mental model-based educational material about workplace PM exposure?’

## Methods

### Materials

We considered six potential concepts for use as an educational material: a folder, a presentation, an instruction movie, an e-learning tool, a serious game, and a practical assignment. We chose these six concepts to accommodate for a large range of options in complexity and scale. We evaluated each of these six options based on the eight criteria mentioned of the SECTIONS model, a framework for selecting an appropriate medium for education developed by Bates & Poole [4]. A detailed evaluation of these six options by Bates & Poole’s [4] criteria, including a table, can be found in Additional file 1.

Based on the SECTIONS model, we decided to develop an educational folder, provide companies with an opportunity to incorporate the folder into a presentation, and amplify the intervention by adding a practical assignment. This way, we cover all of the eight criteria mentioned by Bates & Poole [4] in our intervention. At present, a suitable assignment already exists to be used in practice with minimal adjustments [6]; however, a suitable educational folder still needs to be developed. For that reason, the remainder of this article focuses on the development of the folder.

**Fig. 1:**
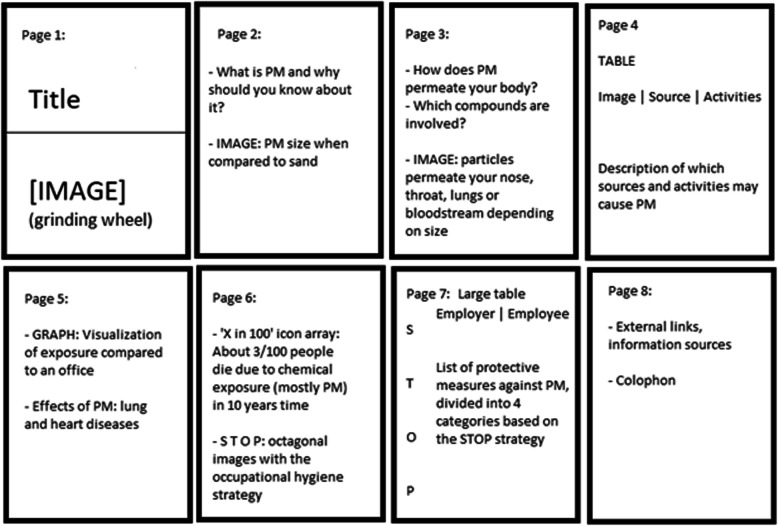
Schematic overview of the folder

When designing an educational folder, the contents should first be decided. Based on our previous study [30], we assessed the information needs of workers in road work companies about the properties, sources and effects of PM, as well as mitigation methods and, wherever possible, the rationale behind them. A schematic overview of the folder we developed can be found in Fig. 1.

Since workers tend to value practical instructions over technical details [24, 25], we minimalized the technical details. Limiting these technical details also helps prevent information overload [9]. For example, we omitted the distinctions between PM_10_, PM_2.5_ and PM_0.1_, which is often used in more academic settings to signify particle size (where PM_10_ refers to particles with a standardized diameter smaller than 10 μm, and so on). In occupational settings, the terms ‘inhalable’, ‘thoracic’ and ‘respirable’ are often used instead of defining particle size by the micrometer, which is more practical, but still has the downside of including technical terminology which may not be necessary. Nevertheless, the distinction between PM that can permeate deep into your lungs and blood stream and coarser fractions of PM that cannot is important. Therefore, we explain in the folder (with an image) on page 2 and 3 that the smaller the particles get, the deeper they can permeate your body.

Page 4 describes sources and activities that may cause PM, and page 5 and 6 visualize the exposure and the risk, respectively. On page 6 and 7 we specifically addressed various mitigation methods using a practical hierarchical model that showcases which mitigation methods should be prioritized (the four-step STOP model; Substitution, Technical measures, Organizational measures and Personal protection) [18]. In order to accommodate for the tensions that may arise between different levels of the company hierarchy (see [30]), the possible mitigation methods were split in two columns, ‘What can the company do’ and ‘What can I do’.

We used the differences between the mental models (see Table 1) to determine which bits of information should be included. For example, we mentioned that PM is usually invisible, due to the fact that it consists of very small particles, and cannot be smelled. We did not include the distinctions between PM fractions such as PM_10_, as mentioned before, but we did mention that black carbon and rubber are important constituents of PM that may cause adverse health effects. We left out any references to ecological effects, since this study is concerned with individual health risks.

**Fig. 2:**
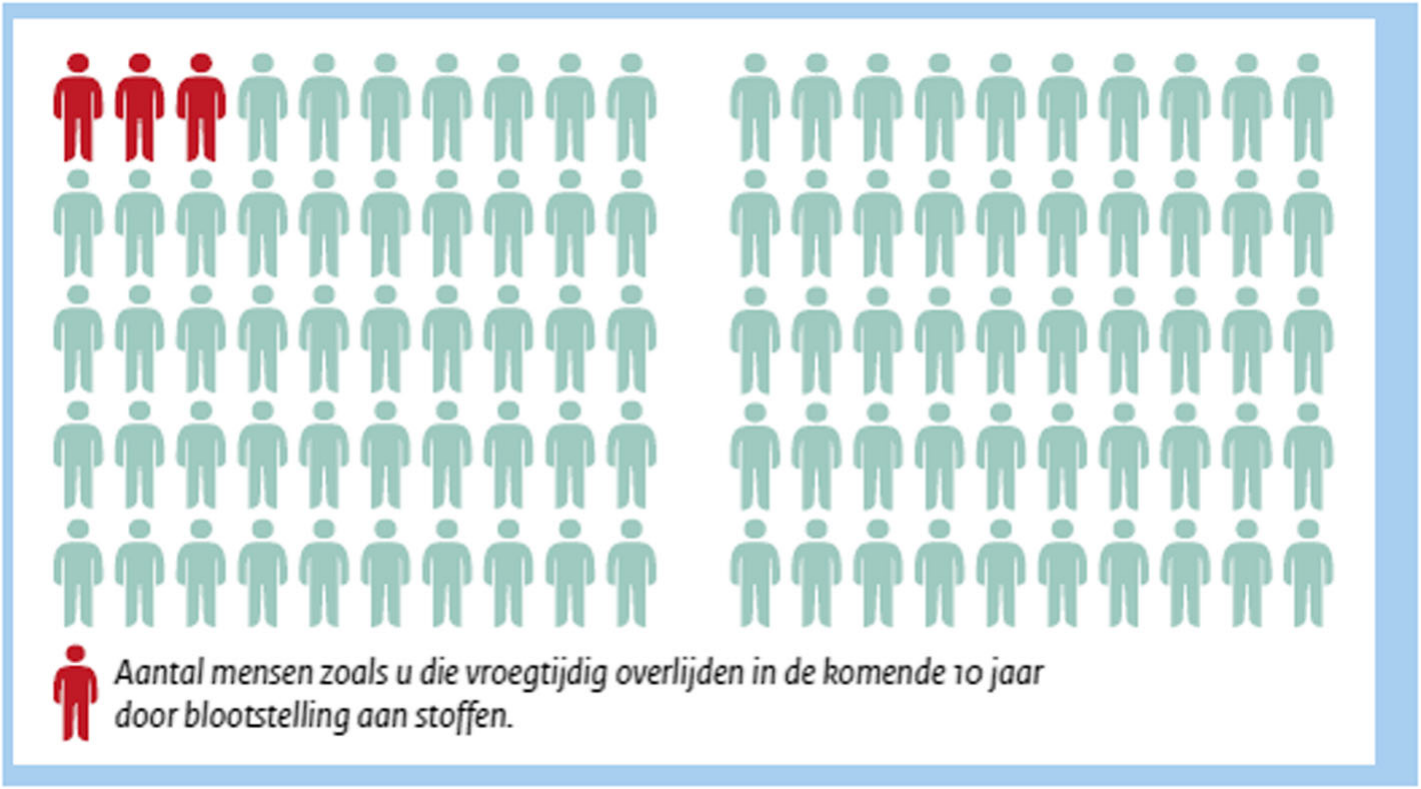
‘X in 100’ graph, as recommended by literature [33, 34, 37]. The text reads (in Dutch): ‘Number of people similar to you that die prematurely in the next 10 years due to chemical exposure.’ The folder goes on to explain that these chemical exposures are mostly PM-related. It should also be noted that this is image does not comply with the normal 10 × 10 standard of the icon array, a choice that was made due to layout issues but was ultimately deemed invalid; it was fixed in a later version (see Fig. 3 in Additional file 2)

Regarding the effects, we mentioned that PM can not only cause lung diseases, but also cardiovascular diseases. We lead with the more well-known lung diseases in the folder, giving examples such as bronchitis and lung cancer, and then we mention that PM may have other effects as well, such as heart diseases. We gave a general indication of the number of deaths per year in the Netherlands, as we felt that this would resonate more with our target group than the number of deaths worldwide. Finally, we included an ‘X in 100’ graph based on the data from a factsheet on chemical exposure in the Arboportaal, the website for Occupational Health and Safety of the Dutch Ministry of Social Affairs and Employment, giving an indication of the chance of premature death for workers exposed to PM (see Fig. 2).

**Fig. 3:**
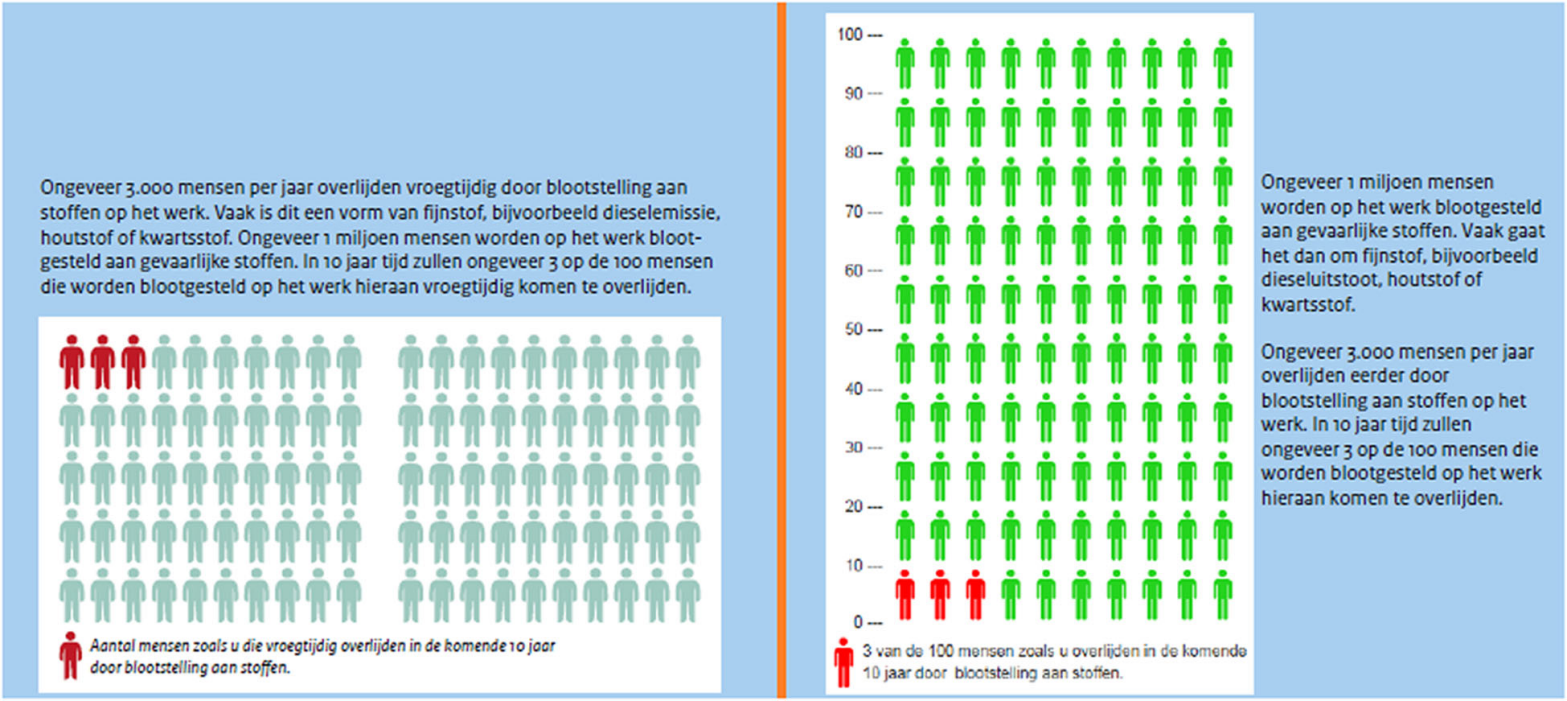
‘3 in 100’ icon array with calculation, in the pre-usability test (left) and post-usability test (right) versions of the folder

### Procedure

In order to investigate the usability of the newly designed educational folder, we contacted four experts and recruited six workers regularly exposed to PM in the workplace for a usability test (more details can be found in Table 3). We conducted a semi-structured interview with each of these participants, face to face and one on one, for ten interviews in total. We developed the interview guideline ourselves, but many of the questions we asked were based on the Suitability Assessment of Materials (SAM) tool [12], which is a tool used primarily to assess the quality of instructions about health related issues for people with low health literacy. The criteria from the SAM involve content, literacy demand, graphics, layout, learning stimulation, and cultural appropriateness [12].

The interviews for the experts started with a few questions where they could specify their daily work and their expertise on PM and risk communication. Subsequently, we asked about the material, starting with a question about the participants’ general impression. We followed with questions about the contents; especially important to us was whether there were any scientific inaccuracies in the folder. We also asked questions about the amount of information, the build-up, the layout, and whether there was enough focus on practical advice, as this is recommended by literature [24, 25]. We also asked how we should cope with potential downsides of our folder, such as the lack of interactivity [4] and the absence of an explicit introduction or conclusion section in our folder [12].

Similar to the expert interviews, the worker interviews started with questions about their daily activities at work, including a question whether they work more inside or outside. This was to get an indication how these workers may be exposed to PM on their job. Beyond that, we asked the workers similar questions about the folder as well, related to the contents, amount of information, build-up and lay-out. We also asked about the workers’ perceptions of PM after reading this folder, including a question whether they were now more inclined to protect themselves, to get an initial indication about the practical applicability of the folder. As always, both interviews concluded with the question whether there was anything else to add. The full interview guidelines, both for the expert and worker, can be found in Additional file 3.

#### Participants

The four experts were all professionals working for health and safety related institutes, and they were recruited from the professional network of the researchers. Two of the four experts worked for the National Institute of Public Health and the Environment (in Dutch: RIVM), one worked for an institute involving agricultural safety, and one worked for the organisation for occupational health and safety in the Netherlands (Arbo Unie). They all had ample knowledge both about PM itself and about risk communication, and they all had at least 10 years of work experience with PM or similar exposure risks as well as a higher education on toxicology, epidemiology or an equivalent study. They were asked for informed consent before the interview, none of them objected to the recording of the interview.

The six workers that may be exposed to PM were recruited from the Flycatcher panel, which is an internet research company from the Netherlands that hosts an ISO certified online panel, for use in studies that require representative samples of participants. To select participants, two questions were asked: ‘Do you work outside or near the side of the road’, and ‘Do you work with machinery or carry out activities such as sawing, drilling or lawn-mowing during your work?’, with the answering options of ‘often’, ‘regularly’, ‘sometimes’, ‘rarely’, or ‘never’. Participants who answered ‘often’ or ‘regularly’ to one or both of the questions and indicated to be interested in participating in an interview were eligible to participate in the interview. This process yielded 39 potential participants with interest in participating. Out of these 39, six participants were selected non-randomly, in order to incorporate various branches, age groups, levels of education, and regions of the country in our sample. These details can be found in Table 2. The participants were asked for informed consent as well as permission for recording the interview.

**Table 2:** Area of work, level of education, region, age and gender of the six participating workers

#	Area of work	Level of education	Region	Age group	Gender
1	Logistics	High	North Holland	60–64	Male
2	Logistics	Low	Gelderland	65+	Male
3	Construction	Medium	Utrecht	30–34	Male
4	Construction	Medium	Gelderland	60–64	Male
5	Agriculture	Low	Overijssel	55–59	Female
6	Construction	Medium	South Holland	35–39	Male

### Analyses and follow-up

After conducting the expert interviews, the first author summarized transcripts and analyzed the interviews in a question-and-answer format. For the expert interviews, we collected all potential improvements to our material suggested by one or more experts into a table (see Table 3). Most of the potential improvements from the expert interviews were straightforward, so we decided to make some adjustments to the folder before starting the worker interviews. Some suggestions, however, proved to be more controversial. One expert thought we should remove the ‘3 in 100’ icon array, because it was based on a calculation that they deemed questionable and confusing. However, two other experts were in favor of maintaining it, praising the insightfulness and the visual appeal of the illustration.

**Table 3:** Experts’ suggestions about a workplace PM exposure folder

Suggested change:	^a^Multiple experts suggest?	Disagreement among experts?	Led to change in folder?
CONTENT			
Clarify that not all types of PM may cause cancer	N	N	Y
Smoking is a source of PM, but should not be in the folder, since it is not work-related	Y	N	Y
Exhaust gases are a source of PM, but should not be in the folder, since it is not work-related	N	N	N
Legal exposure limits should be included	N	Y	N
Mention that not only peak exposure, but also overall exposure is important	N	N	Y
Maintain the calculation that shows ‘3000 people dying as a result of PM’, but make sure it does not cause confusion	Y	N	Y
LITERACY DEMAND			
Use simple language and remove complicated idioms	Y	N	Y
Use nuanced and objective language	Y	N	Y
GRAPHICS			
Remove the ‘X in 100’ array	N	Y	N
LAYOUT			
The order of the folder should start with the measures against PM	N	N	N
Have an introduction section and a conclusion	N	N	N
LEARNING STIMULATION			
Clarify that there are various types of respirators, not all of which are effective against PM	N	N	Y
‘Process automatization’ should be mentioned as a measure against PM	N	N	N
‘Vacuum cleaning instead of sweeping or using compressed air’ should be included as a measure against PM	N	N	Y
Sweeping the floor should be mentioned as a cause of PM	N	N	N
Provide a reference to a common VEM system (Video Exposure Monitoring) at the back of the folder^b^	N	N	Y
Remake the folder into a collection of separate elements which can be combined by a professional, to be more tethered to a specific target group	N	N	N
CULTURE			
Show types of jobs with high PM exposure, instead of just tasks	N	N	N

^a^Legend: The column ‘Multiple experts suggest’ signifies whether or not a particular recommendation was done by more than one of the four participating experts in this study. The column ‘Disagreement among experts’ signifies whether or not a particular recommendation by one expert was explicitly disapproved of by another expert. The column ‘Led to change in folder’ signifies whether or not we made any changes to the folder based on a particular recommendation. (Y = Yes, N = No)

^b^VEM or Video Exposure Monitoring is a movie-based method of instruction, in which people are shown doing work in ways that cause high and low levels of exposure to a certain agent [5].

**Table 4:** Evaluation of the six potential educational materials by means of the SECTIONS model

Criteria	Folder	Presentation	Movie	E-learning	Game	Assignment
Appropriateness for target audience (‘Students’)	+	–	+	–	–	+
Ease of use	+	+	+	–	–	–
Costs	+	+	–	–	–	–
Appropriateness for the learning goals (‘Teaching’)	–	–	+	+	–	+
Interactivity	–	+	–	–	+	+
Organizational issues for implementation	+	+	+	–	–	–
Novelty	–	–	–	+	+	+
Speed of development & revision	+	+	–	+	–	+

The worker interviews were analyzed in the same way as the expert interviews. Since there were not nearly as many suggested improvements by the workers, we decided not to assemble these in a table, but instead opted to show some of the most relevant quotes from these interviews, as is good practice in qualitative research.

## Results

### Recommendations from expert interviews

The four experts gave a large number of recommendations for the folder, most of which were relatively minor. The results of these interviews will be presented in the form of a table (see Table 3), and classified within the six main categories of the SAM tool [12]: content, literacy demand, graphics, layout, learning stimulation, and cultural appropriateness. Minor adjustments, such as changes of a single word or phrase, are not shown in this table. Some suggestions were done by several experts, and these always led to a change in the folder; other suggestions by one expert were explicitly disagreed with by another expert, and these suggestions never led to a change in the folder. However, all suggestions, including these, were evaluated on a case-by-case basis. This can also be seen in more detail in Table 3.

### Recommendations from worker interviews

Contrary to the four experts, the workers had few comments about the folder. When asked what their general impression was, most workers responded that they were satisfied with how the folder looked, although some of them still made a few suggestions for improvement. Some more detailed descriptions of participants’ views will be outlined below, and they will again be classified within the six categories of the SAM tool [12].

### Content

One participant stated that wood particles should be mentioned as a source of PM somewhere. One participant thought that a recommendation to work night shifts should not be in the folder, since it would lead to other adverse health effects. Another recommendation would be to add a clarification how long respirators can be used:

*For how long can you use a respirator? It is never stated on the thing itself. I always wait until it looks dirty, [ … ] but then I am too late already. [worker 3]*

Based on these comments, we removed the recommendation to work night shifts, and we added wood particles as one of the forms of PM near the ‘3 in 100’ icon array, as wood particles are also mentioned in the source material. As there is no clear-cut answer to the question how long respirators should be used, we would have needed to expand the folder quite a bit to address this issue thoroughly enough; we decided against this to maintain its brevity.

### Literacy demand

The participants had no clear problems regarding the understandability of the text. One interesting problem was found in the four-step occupational hygiene strategy, regarding the word ‘Substitution’, of which at least one participant did not know the meaning. However, since this word is essential in maintaining the four-letter STOP strategy for occupational hygiene, we kept it in. Otherwise, no problems with the difficulty of the text were identified. Several participants correctly identified that PM is invisible, and that its health effects may include not only lung diseases, but also cardiovascular diseases.

Some of the phrases in the folder inadvertently led to misconceptions. For example, one participant thought that three in hundred workers who are exposed to PM acquire adverse health effects, which indicates that they took the ‘3 in 100’ icon array to mean something different than intended. One other participant correctly identified that the ‘3 in 100’ refers to deaths, but thought that only 3 in 100 died of PM exposure in their lifetime, even though it is meant to refer to the next 10 years. We discuss the issues with the icon array further in the discussion section, as well as in Additional file 2.

### Graphics

Regarding the layout, participants thought that the folder looked professional and that the illustrations helped to maintain the interest of the reader. Therefore, no changes to the graphical layout were made. Worker 4, who contrasted our folder with an existing folder about PM and pneumoconiosis, stated that ours avoids the mistake of overwhelming the reader, for example with a picture of a dying man with a severe lung disease:

*One of my colleagues died in 2014, lung cancer due to asbestos.[ … ] If you look at the hospital bed, you are reminded of that thing [company name] gave us [because it shows a dying man due to pneumoconiosis]. [ … ] I do not like what they are doing; they should not do that. [ … ] You should not see someone die like that. I find this much more appealing and a better explanation. [worker 4]*

### Layout

Two separate workers, both of whom were above 60 years of age, thought that the font was too small. Therefore, we increased the font size of the folder (see also Additional file 2). Otherwise, no further comments regarding the layout were found.

### Learning stimulation

One participant was not quite sure whether the amount of information in the folder was adequate, and that it may be either too little or too much depending on the situation in which it would be used. On the other hand, one participant specifically praised the amount of information, and contrasted it with an existing, much more elaborate folder about PM and pneumoconiosis:

*It is just fine. No more is needed. [ … ] You can give a lot more information, if you are sawing than you need to do this, but [everything you need] is in here. [Company name] gives way too much information, that is their down side. [worker 4]*

### Culture

Several participants asked whether the folder was going to be translated in other languages, specifically languages of common minority groups in the Dutch workforce. This is illustrated by the following quotes:

*Will it also be in other languages? Because there are always a lot of Polish guys working there. [worker 1]*

*Foreigners, they do not know anything about this. So you should translate this into ten different languages. [worker 2]*

At present, no foreign language versions of the folder exist, but this may change in the future.

## Discussion

In this article, we discussed the development of an educational material to provide workplace risk communication on PM exposure risk, and we asked the question to what extent experts and workers would value the usability of this educational material in the workplace. We investigated various methods of instruction, assessed a combination of a folder embedded in a company training with a practical assignment to be the best fit, and subsequently developed the folder based on our earlier findings from our mental models study [30]. The resulting folder was presented to ten stakeholders – four experts and six workers that may be exposed to PM – which led to a considerable amount of commentaries and suggestions. More details on the adjustments based on these suggestions can be found in Additional file 2.

### Strengths and limitations

Reflecting on the process by which we developed the folder, it is noteworthy that we used a combination of various models and methods. A strength of combining these models and methods is the way in which they supplement each other. We used the mental models approach to identify stakeholder information needs, in order to provide appropriate information in the folder from the user’s perspective [23, 26, 29]. For example, the fact that PM is mostly invisible and the fact that it may cause cardiovascular diseases are often unknown to workers [30], so we included these bits of information in the folder to alleviate common misconceptions. The protection motivation theory, or PMT [27], then plays a role in ordering and structuring the information. We present facts about PM such as its invisibility and its tendency to cause (cardiovascular) diseases first, in order to increase threat appraisal; then, near the end of the folder, we thoroughly discuss prevention methods in order to increase coping appraisal. This focus on coping appraisal is especially important within the framework of the PMT, because only focusing on threat appraisal may be counterproductive for improving safety behavior [16, 28].

The mental models approach and the PMT gave us insights in which information should be presented, but the SECTIONS model [4] answered the question how it should be presented. From six potential concepts of an educational material, we eventually chose two that may be combined, namely the folder and the practical assignment. We chose for these two options, as the positive and negative aspects of these concepts appear to balance each other out, and the two concepts appear to be most suitable for our situation; furthermore, the practical assignment makes the health threat we discuss in the folder more tangible by means of active learning [7]. The folder was filled in with the information as determined by the mental models approach and the PMT.

Finally, we kept the principles for designing educational material for low health literacy people in mind as mentioned by the Suitability Assessment of Materials (SAM) tool [12], and we also used this SAM tool for a formative evaluation within the context of the usability test in this study. After combining these methods and theories to design and develop our folder, we tested and adjusted the layout and contents of the folder based on comments by both professional occupational hygienists and workers in professions involving particulate matter. The method we used to develop this folder resulted in a product that was generally viewed as favorable by the participants in this study. This can be seen by the nature of their suggestions, as they mostly suggested minor alterations and clarifications, while the main contents, layout and structure were often praised.

Nevertheless, the method we used has its limitations. There is no theoretical basis to assume that the different models can be integrated. The SECTIONS model itself is not based on evidence from learning and behavior theories, but experience-based and highly subjective; this is acknowledged by the authors, who argue that “*decision making in this area cannot be driven by hard and fast formulae or rules”* [4]. Indeed, a more scientific approach to this end does not appear to exist. However, we found that making use of these models supplements the mental models approach, as the mental models approach mostly answers questions related to the content of the material, whereas the shape and the usability of the material are equally important.

A limitation of the usability test itself, of course, is the small sample size. We only included a small number of participants in this study so that we could focus on the design and the development of the folder before testing it in a larger-scale experiment. In most cases, we were able to find a consensus on the preferred design of the folder. However, this was not the case for the risk visualization, as experts strongly disagreed among each other whether the ‘3 in 100’ icon array should be used in our folder. The visualization itself was commended by two of the four experts; however, the calculation on which the image was based was seen as vague and even potentially misleading. Indeed, direct data on worker deaths due to PM was unavailable, and we used data from all chemical exposure (including but not limited to PM) instead, but we clarified this in the folder. Interestingly, another issue with the icon arrays was found in literature, as studies disagree whether this type of visualization is sufficiently effective [14, 20, 34]. Overall, the question whether the icon array should be included in our folder remains unresolved, and more research on this subject is needed.

Finally, one limitation of this study is the focus on the folder. In the Method section of this article we described a design involving both a folder and a practical assignment. We are aware that the usage of the folder alone may not result in the outcomes of awareness and attitude that we would like to achieve. Nevertheless, as mentioned, an assignment from an earlier study [11] can likely be reused with minimal changes, and we will study the combination of such an assignment and our folder in future research.

One downside that cannot be alleviated is costs. A work safety meeting that involves our proposed design consists of four steps: filling out a pretest questionnaire, reading a folder, fulfilling a measurement assignment at the workplace and filling out a posttest questionnaire. The time constraint easily adds up to half an hour on the folder and half an hour for the measurement assignment, and on top of that half an hour for discussing and explaining the measurements. Furthermore, the measuring equipment invokes considerable financial costs. Nevertheless, we found in our earlier study [30] that work safety meetings that consist of only reading material are heavily criticized and appear not to be very effective. Therefore, we will test the combined method of folder and measurement assignment and will add a reflection of the total costs and benefits.

## Conclusion

As a result of this study, we deemed the updated version of the folder fit for a larger scale experiment. In this experiment, we will test the learning effects of our folder. We will also investigate whether our newly developed folder has any added effect compared to existing material, when evaluated on PMT-related outcomes of threat appraisal, coping appraisal and safety behavior. Since the ‘3 in 100’ icon array has proven to be controversial, there will be two versions of this folder, one with and one without the icon array. We will test both of these versions against each other and against an existing folder in a digital experiment, and we will include a control condition with an unrelated text. After further studying the quality of the folder, we proceed by testing the folder alongside the assignment at the workplace. This practical test will investigate the added effects of the assignment as opposed to only using the folder, and it has the additional benefit of testing the folder in a practical setting.

## Supplementary Information

**Additional file 1.** Evaluation of six potential materials by means of the SECTIONS model [4].

**Additional file 2.** Most important changes to the folder based on the interviews.

**Additional file 3.** Interview guideline for expert and worker interviews.

## Data Availability

The data are not found online, but can be obtained from the lead author upon request.

## Abbreviations

ISO: International Organization for Standardization
PM: Particulate Matter
PMT: Protection Motivation Theory
RIVM: Rijksinstituut voor Volksgezondheid en Milieu (National Institute for Public Health and the Environment)
SAM: Suitability Assessment of Materials
SECTIONS: Students, Ease of use, Costs, Teaching, Interactivity, Organizational issues, Novelty & Speed
SPR: Strategic Programme RIVM
UMC: University Medical Center
VEM: Video Exposure Monitoring
WHO: World Health Organization
WMO: Wet Medisch wetenschappelijk onderzoek met mensen (Medical Research Involving Human Subjects Act)
