# Supplementary material for: Development and usability of educational material about workplace particulate matter exposure
Source: BMC Public Health. 2021 Jan 22;21:198. doi: 10.1186/s12889-021-10197-x (PMC7821551; doi:10.1186/s12889-021-10197-x)
Supplement: Supplementary file 1 — Additional file 1. Evaluation of six potential materials by means of the SECTIONS model [4]. [file 12889_2021_10197_MOESM1_ESM.docx]

**Additional file 1: Evaluation of six potential materials by means of the SECTIONS model (Bates & Poole, 2003).**

The eight criteria in the SECTIONS model include: Ease of use, costs, appropriateness for the students, appropriateness for the learning goals, interactivity, organizational issues towards implementation, novelty, and speed (both speed of development and of revision). We will evaluate the six approaches we considered – a presentation, a folder, a movie, an E-learning system, a serious game, and a practical assignment – based on these eight criteria. The results can be found in Table 4. We will then elaborate on the choices we made in our evaluation, and discuss some other considerations before we made the final decision as seen in the main text of this article.

| *Criteria* | Folder | Presentation | Movie | E-learning | Game | Assignment |
| --- | --- | --- | --- | --- | --- | --- |
| Appropriateness for target audience (‘Students’) | + | - | + | - | - | + |
| Ease of use | + | + | + | - | - | - |
| Costs | + | + | - | - | - | - |
| Appropriateness for the learning goals (‘Teaching’) | - | - | + | + | - | + |
| Interactivity | - | + | - | - | + | + |
| Organizational issues for implementation | + | + | + | - | - | - |
| Novelty | - | - | - | + | + | + |
| Speed of development & revision | + | + | - | + | - | + |

*Table 4.* Evaluation of the six potential educational materials by means of the SECTIONS model.

**Students:** An important issue in our situation is involving several types of workers in our intervention. There are large differences in literacy and level of education among workers. A presentation tends to have the downside of mostly answering the questions of those who already had some basic knowledge; e-learning systems and serious games may not be suitable for less educated employees. The remaining three options appear to have the highest chance of involving everyone.

**Ease of use:** The more complicated options – a serious game, an assignment and an e-learning system – all need a briefing or some practical instruction for the teacher in order to succeed. The other three options are more straightforward to use.

**Costs:** Developing a movie, e-learning system or serious game is very time-consuming and therefore expensive. A practical assignment is quicker, but measuring equipment can still drive up the costs.

**Teaching/learning:** A game tends to have many superfluous bits of information to the learning goal. Presentations and folders often make it difficult to maintain the learner’s attention. The other three options are generally clear and interesting to the learner.

**Interactivity:** Interacting with a folder, movie or e-learning tool is not as easy as with a teacher giving a presentation, explaining the rules of a serious game, or giving an assignment.

**Organization:** A folder, a presentation and a movie can be implemented in an existing workplace situation more easily than the other three options.

**Novelty:** E-learning tools, games and assignments are not often used as a means of risk communication in workplaces yet, and therefore they are interesting for the novelty factor.

**Speed:** Adapting a movie or a game is extremely time-consuming; the other options are clearly more suitable for a ‘rapid prototyping’ idea.

*Other considerations.* The first option, a folder, is fairly easy to create based on our mental models research, and also easy to implement, since we had found that many companies already use similar materials for other safety-related subjects. On the negative side, however, a folder lacks interactivity, might not be sufficiently stimulating and thus not fulfill its learning goals, and it is not very original. Compared to a folder, a presentation is similar; it is more interactive than a folder, but it might not be as appropriate for the learners as they do not always have a solid reason to pay attention. A movie may be a lot more stimulating, but it is not very cost-effective. E-learning and serious games are useful educational tools, but we deemed them unfit for our current situation as they have too few upsides for a small-scope intervention such as this.

Finally, a practical assignment in the workplace, for example using a PM meter, is also viable option. For example, participants could use a PM meter with display, looking for the occupational activity or microenvironment generating the lowest and highest exposure. A real life measurement assignment such as this can be seen as a form of active learning (Bonwell & Eison, 1991), and thereby stimulates thinking. Moreover, it forces the participants to go over all activities and think about the exposure. Additionally, it may introduce a gaming element in which the group of participants is battling for the lowest and highest measurement results. In earlier research, we used wearable sensors to determine workers’ exposure to PM and other agents, and gave them feedback by means of a presentation (Bolte et al., 2018). This sparked discussion about safety behavior, both work-related (mowing downwind) and not work-related (smoking). An assignment involving PM meters and magnetic field exposimeters has also been used before (Den Broeder & Bolte, 2018). In this earlier study on environmental exposures in an occupational setting with 40 higher educated professionals, it was shown that finding the highest and lowest exposure situations and discussing it in the group made them assess their environment seriously. These promising results may be extrapolated to our current situation.
