# Supplementary material for: Development and usability of educational material about workplace particulate matter exposure
Source: BMC Public Health. 2021 Jan 22;21:198. doi: 10.1186/s12889-021-10197-x (PMC7821551; doi:10.1186/s12889-021-10197-x)
Supplement: Supplementary file 2 — Additional file 2. Most important changes to the folder based on the interviews. [file 12889_2021_10197_MOESM2_ESM.docx]

**Additional file 2: Most important changes to the folder based on the interviews**

We will discuss the most important findings and changes to our folder from the interviews, in accordance with the six categories of the SAM tool: content, literacy demand, graphics, layout, learning stimulation, and cultural appropriateness (Doak et al., 1996). However, no changes related to culture were made in our folder, so five of these categories remain.

*Content*

Some of the wordings were changed after the expert interviews, in order to accommodate for the fact that PM does not always cause cancer and that overall exposure is just as important to mention as peak exposure. We also made a number of changes based on the worker interviews, for example because working night shifts might not be ideal as a mitigation method, since it introduces new safety risks. For that reason, we removed any literal reference to night shifts, instead saying more generally that employers may adjust work schedules to combat PM exposure. We also added some information about the type of respirator that should be used.

*Literacy demand*

The folder was rechecked for language issues after some of the experts mentioned issues with difficulty and neutrality. Accordingly, we removed difficult wordings such as ‘prematurely deceased’, and we changed contentious phrases such as the description of PM as an ‘assassin’ to a more nuanced statement such as ‘PM is mostly known for its long-term effects’.

*Graphics*

The most noticeable change in graphics can be seen in the section with the ‘3 in 100’ icon array and the corresponding calculation. This may have been the most controversial part of the folder, with two experts claiming that the calculation was confusing and one expert insisting that the icon array should be removed altogether. Furthermore, various workers found the section to be confusing as well. We decided not to remove it, in order to stay in line with the theoretical background (Trevena et al., 2013), but we did overhaul the section to make both the image and the calculation clearer. The result can be seen in Figure 3.

*Layout*

Two separate workers mentioned that the font size was difficult to read for elderly people. In order to accommodate for this, we increased the font size by one point, and moved around some of the text to make sure that everything would still fit. A higher increase in font size would mean that we had to add more pages or remove information from the folder, both of which we deemed unacceptable.


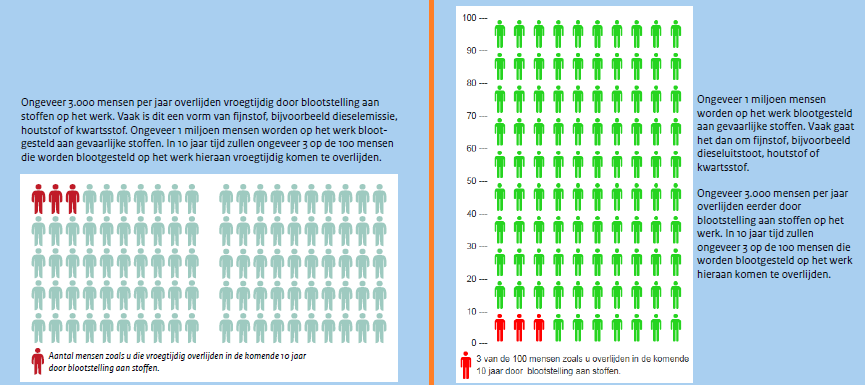


Figure 3. *‘3 in 100’ icon array with calculation, in the pre-usability test (left) and post-usability test (right) versions of the folder.*

*Learning stimulation*

The workers’ comments about the amount of information, that may either be too high or too low depending on your specific purposes, did not lead to any further changes. The main reason for this is that our folder occupies a niche that is not yet occupied by any other material, and any more in-depth ideas can always be shared in a work safety meeting alongside the folder.
