# Supplementary material for: Development and usability of educational material about workplace particulate matter exposure
Source: BMC Public Health. 2021 Jan 22;21:198. doi: 10.1186/s12889-021-10197-x (PMC7821551; doi:10.1186/s12889-021-10197-x)
Supplement: Supplementary file 3 — Additional file 3. Interview guideline for expert and worker interviews. [file 12889_2021_10197_MOESM3_ESM.docx]

**Additional file 3: Interview guideline for expert and worker interviews**

*Expert interview*

- [Welcome; Informed consent]
- Before we start talking about the folder, could you tell me something about your work / your expertise?
  - How many years of experience do you have?
  - What was your prior education?
- What was your general impression about the educational material?
  - [We show our material step by step and discuss the following aspects:]
    - [Design choice]
    - [Content selection: background, PM sources, health effects, measures]
    - [Mental model subjects such as: visibility, cardiovascular disease…]
    - [Illustrations, especially the visualizations of risk]
- What is your opinion about the material’s contents?
  - Have you identified any factual errors? If so, could you elaborate?
  - What is your opinion about the amount of information?
    - Do you have any suggestions about which information to add or to remove?
  - How do you feel about the structure of the folder?
- Our main end users of the folder would be practically educated employees working in the roadwork and construction branches. To what extent do you feel that this material fits the end users?
  - Could you elaborate on language use?
  - Could you elaborate on the appropriateness and content of the illustrations?
  - Can you tell something about cultural factors?
- We wished to put a focus on practical instructions. To what extent do you feel that we have succeeded in doing so, and how could we do any better?
  - How would you describe the balance between what the company could do and what the employees could do?
  - To what extent are our recommendations viable?
  - How could we include any further means to help employees protecting themselves?
- What is your opinion on the layout of the folder? (For example, note the positions of illustrations and text, whitespace, color use, contrast, line length…)
- Our folder is not very interactive. How could we accommodate the need for interactivity in work safety meetings, if needed and possible?
- We chose not to include a lengthy introduction or conclusion, in order to improve the folder’s concision. How do you feel about this choice?
- Which information in the folder would you classify as essential core information?
- Which information may be less essential or even superfluous?
- Are there any campaigns or other events going on right now that are relevant for us to keep into account?
- Are there any other things you would like to discuss?

*Worker interview*

- [Welcome; Informed consent]
- Before we start talking about the folder, could you tell me something about your work?
  - Could you describe an average working day?
  - Do you mainly work indoors or outdoors?
  - How many years of experience do you have?
- What is your opinion on this folder?
  - What is your opinion about the amount of information?
  - How do you feel about the structure of the folder?
  - What is your opinion on the layout of the folder? (For example, note the positions of illustrations and text, whitespace, color use, contrast, line length…)
- Can you describe in your own words what this folder is about?
  - Can you explain what PM is?
  - Can you tell me something about the sources of PM?
  - Can you tell me something about the effects of PM exposure?
  - Can you explain what to do against PM?
    - Which measures against PM did you already take?
    - Which measures against PM would you be less likely to take?
    - Would you become more or less likely to take certain measures against PM after reading this folder?
- How do you see your own PM risk right now?
- To what extent do you feel that this folder is suitable for workers in your situation?
  - To what extent does this folder fit your situation?
- To what extent is a folder a suitable material for explaining PM risk?
  - Do you have any suggestions for other materials?
- Which measures against PM are being carried out in your workplace?
  - How did your employer educate you on PM?
- Are there any other things you would like to discuss?
